# Global Gene Expression Analysis in an *in vitro* Fibroblast Model of Idiopathic Pulmonary Fibrosis Reveals Potential Role for CXCL14/CXCR4

**DOI:** 10.1038/s41598-018-21889-7

**Published:** 2018-03-05

**Authors:** Luis R. Rodriguez, Margaret Emblom-Callahan, Mantej Chhina, Sarah Bui, Bilal Aljeburry, Luc H. Tran, Rebecca Novak, Merte Lemma, Steven D. Nathan, Geraldine M. Grant

**Affiliations:** 1 Department of Biology, George Mason University, 10900 University Blvd., Manassas, VA 20110 USA; 2 Inova Advanced Lung Disease and Transplant Program, Inova Heart and Vascular Institute, 3300 Gallows Road, Falls Church, VA 22042 USA

## Abstract

Idiopathic Pulmonary Fibrosis (IPF) is a progressive disorder that is marked by an over accumulation of activated fibroblast populations. Despite the improved understanding of many mechanisms within this disease, global gene expression analysis has few focused studies on the fibroblast, the central effector cell of progressive fibrosis. We present a unique analysis of IPF pulmonary fibroblasts as they transition through cell culture and identify *in vitro* altered cellular processes. Fibroblasts were isolated from diseased (n = 8) and non-diseased (n = 4) lungs. Global gene expression analysis was carried out at the initial point of isolation and after 3 weeks of culture. We identify several genes that are altered by removal of the fibroblast from the IPF environment. Comparison of this subset of genes to four previously published whole lung analyses refined our list to a small subset of key fibroblast specific genes important in IPF. Application of STRING database analysis and confirmation via *in-vitro* and histological assay highlights the CXCL14/CXCR4 chemokine axis with a possible role in the progression and/or activation of fibroblasts within the IPF lung. Our findings, present a possible therapeutic target for IPF and a model for the study and discovery of novel protein and processes in this terrible disease.

## Introduction

Idiopathic pulmonary fibrosis (IPF) is a lethal form of interstitial lung disease of unknown etiology. IPF has been defined as a disease of unregulated wound repair. This is likely driven by an abnormal epithelium and propagated by a dysregulated overabundant, heterogeneous, fibroblast population in various states of activation^1–5^. The overabundance of these cells and their excessive extracellular matrix (ECM) deposition aids in the progressive fibrosis observed. This results in tissue distortion, impaired gas exchange, and ultimately organ failure^1,6^. The exact source of the IPF fibroblasts population remains unclear, however there are four potential reservoirs, including resident lung fibroblasts, circulating fibrocytes, epithelial cells that have undergone epithelial-mesenchymal-transition (EMT), and pleural mesenchymal cells^7–9^. As the fibroblast is so integral to the pathogenesis of IPF, much effort has been directed toward characterizing the specific processes within this cell. However, a comprehensive global understanding of the IPF fibroblast remains elusive.

Investigation of this disease using *in vitro* fibroblasts as a model system has revealed many important IPF mechanisms and molecules including transforming growth factor beta (TGF-beta1), tumor necrosis factor, and mediators of the Wnt pathway^10–15^. Despite this improved understanding of possible pathogenic mechanisms of IPF, only two therapeutic options have emerged each of which serve to slow disease progression. Global gene expression analysis is a powerful method for investigating the transcriptome and cellular diseased states, however analysis of isolated IPF and normal fibroblasts has shown no discernible genomic differences between populations^10,16–18^. Some data exists on the global genomic phenotype of IPF whole lung tissue in comparison to hypersensitivity pneumonitis, non-specific interstitial pneumonitis (NSIP) and emphysema. In addition, differential gene expression in familial and sporadic interstitial pneumonia has also been described^19,20^. However, the use of whole lung tissue in these experiments does not allow for characterization of the specific cellular contributions to disease.

The pace of progress is hampered in part by the imperfections of *in vitro* models of disease; specifically the observation that once primary cells such as fibroblasts, chondrocytes, melanocytes, and hepatocytes are placed in tissue culture they undergo phenotypic changes affecting biological processes, cell motility, cytokine production, and cytoskeletal arrangements^21–28^. These changes often result in the primary cells bearing little resemblance to their cells of origin.

We have previously reported the development of a method for the isolation of IPF and normal fibroblasts (IPF-F and normal-F) directly from lung tissue without the confounding effects of tissue culture. This method revealed over 1700 significantly differential expressed genes between normal-F and IPF-F^17^ at the time of isolation. Here we observed and analyzed these cells after three passages (P3) in culture. We expanded our analysis to include our initial list of 1700 differentially expressed genes and four previously published lists that identified differences in genomic expression between IPF and normal whole lung isolates^29–32^. These data revealed that through culture the genomic profiles of these cells change *in vitro* and we hypothesized that characterization of the adaptive processes evident in the transition to *in vitro* culture may enable the identification of novel IPF related mechanisms or proteins that may be suitable for therapeutic intervention.

## Results

### IPF and Normal fibroblast *in vitro* demonstrate no statistically significant differential global gene expression profile

We have previously demonstrated that IPF-F and normal-F have significantly different global gene expression patterns when freshly isolated^17^. However, in this investigation we found that once these cells transition to the *in vitro* state, this differential profile is lost. Specifically, gene expression analysis, using Statistical Analysis of Microarray (SAM (FDR = 0%; delta = 0.30)) software, of cultured IPF-F (n = 8) and normal-F (n = 4) demonstrated no statistically significant differential gene expression between these two populations.

### IPF and Normal fibroblasts individually undergo significant gene expression change as they adapt *in vitro*

**Figure 1:**
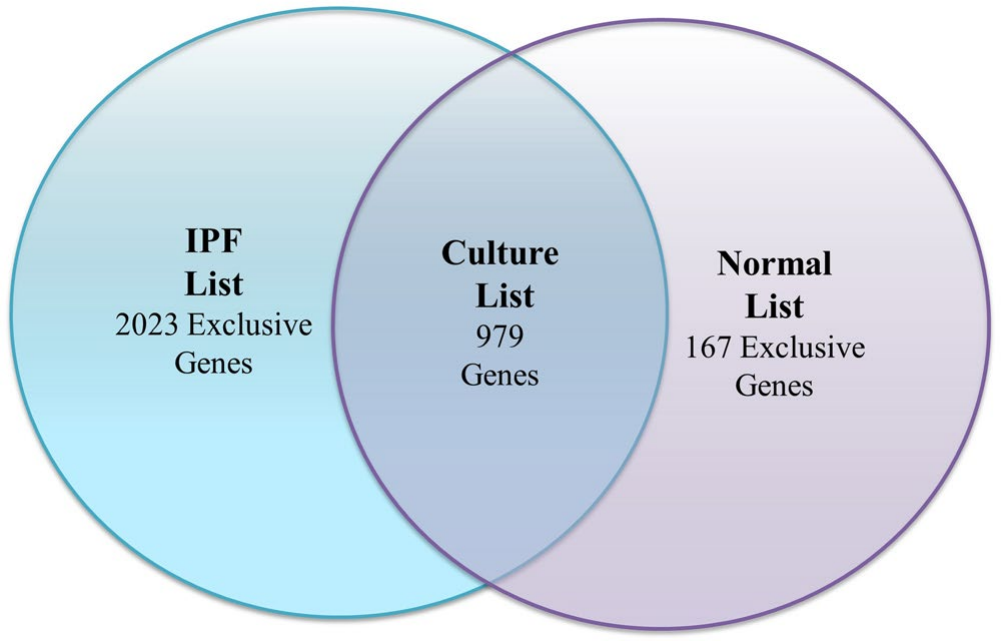
Total number of significant gene expression changes in IPF-F and Normal-F. Graphical representation of the overlap of gene expression changes that occur in IPF-F and normal-F. The IPF List (Table S1) includes 2889 genes in total that change significantly *in vitro*. The normal list (Table S2) contains 1118 genes that change significantly *in vitro*. The overlap between these two lists (979 genes) represent a cluster of genes that is altered significantly within both fibroblast populations and is termed the Culture List (Table S3). Genes outside of the overlap show differential gene expression as a result of culture that are exclusive to IPF-F or normal-F.

Analysis of the gene expression changes that occur in IPF-F (n = 8) and normal-F (n = 4) from initial isolation (non- cultured) to 3 passages *in vitro*, revealed 2889 and 1118 genes to be significantly altered, respectively (Fig. 1).

**Figure 2:**
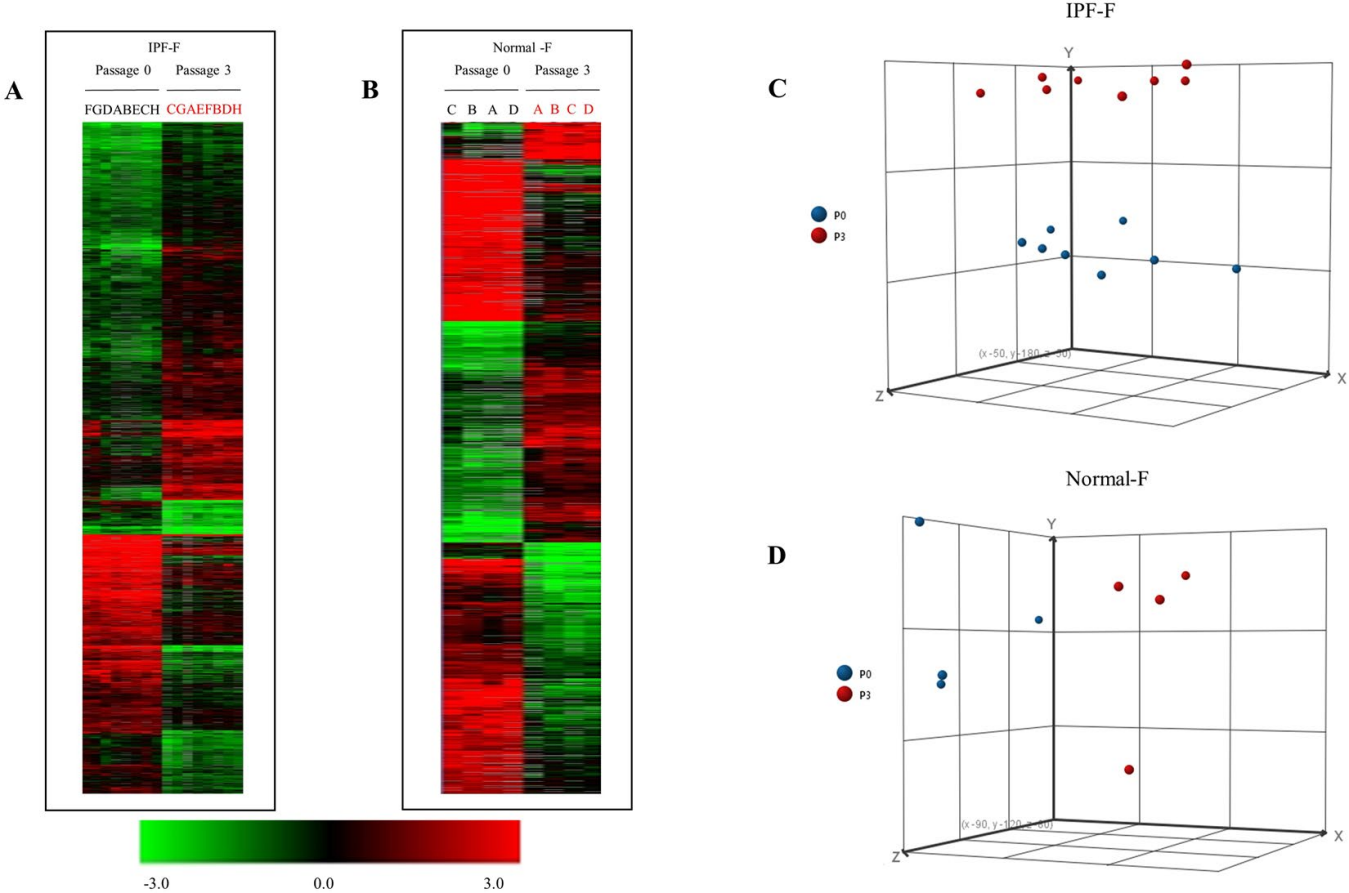
Heat Maps for Significant Gene Expression changes through culture in IPF-F and Normal-F. Hierarchical clustering of significant gene expression changes that occur because of culturing IPF-F and normal-F *in vitro*. Samples are in columns, genes in rows. Those samples denoted in red are fibroblasts that were cultured for three weeks prior to the isolation of RNA, those in black were non-cultured (P0). (**A**) IPF-F lines at P0 (n = 8) and the corresponding P3 (n = 8) were analyzed. (**B**) normal-F lines at P0 (n = 4) and the corresponding P3 (n = 4) were similarly analyzed. (**C**) PCA analysis of the IPF-F demonstrates two clusters corresponding to each time point P0 and P3. (**D**) PCA analysis of the Normal-F demonstrates a similar pattern of clustering based on P0 or P3 grouping. These samples fall distinctly into two clusters P0 and P3, which represent the significant and global changes that occur as the cell populations adapt to the *in vitro* environment. The complete gene lists with log ratios is available in Tables S1 and S2.

Hierarchical clustering (Euclidian distance) and principle component analysis demonstrated clear clustering and separation between the non-cultured fibroblasts (P0) and the *in vitro* (P3) fibroblasts for both normal (Fig. 2B,D) and IPF populations (Fig. 2A,C). This highlights the significant distinction in the global gene expression before and after cell culture. The popular explant outgrowth model system does not allow for analysis of the fibroblast transcriptomes prior to the 6–8 weeks of culture required to develop the fibroblast population. The uniqueness of our model is the insight into this 6–8 week window that enables the delineation of genomic transition i*n vitro*.

Analysis of the fold change in genes associated with myofibroblast differentiation and subpopulations reveals that the IPF-F and normal-F adapt to tissue culture by the promotion of markers typically associated with fibroblast activation such as ACTA2, CTGF, FGFR1 (Table 1). However, the majority of these changes in the normal-F population did not reach statistical significance due to the small sample size. It is of interest to note that the changes (increase/decrease) in gene expression for these select genes tend to be in the same direction for both IPF-F and normal-F. This highlights the environmental impact of the tissue culture model on the fibroblast independent of disease state.

**Table 1:** Fold Change in Select Genes Associated with Myofibroblast Subpopulations.

Gene Name	Gene Symbol	IPF-FC	Normal-FC
Alpha Smooth Muscle Actin	ACTA2	2.424*	5.704
Peroxisome proliferator activated receptor gamma	PPARG	0.197*	0.576
Thy-1 membrane glycoprotein	THY-1	2.669*	15.183*
S100 calcium-binding protein A4	FSP1	0.394*	0.374
Connective tissue growth factor	CTGF	3.101	4.348
TGF-beta receptor type 1	TGFBR1	1.154	1.516
Collagen alpha 1(I)	COL1A1	0.969	2.803
Transcription factor 19	SC1	1.766	1.659
Fibroblast Growth Factor Receptor 1	FGFR1	2.353	2.768

Changes in gene expression of genes typically selected to identify subpopulations of myofibroblasts from heterogenous populations. Statistically significant fold changes are denoted with an * as determined by SAM (FDR = 0%; delta = 0.30).

### IPF specific *in-vitro* adaptation is enriched for mitochondrial and ER related processes

The subset of 1864 gene transcripts which were significantly altered in IPF-F only, as they adapted to the *in vitro* environment, were further analyzed for function using the Database for Annotation, Visualization and Integrated Discovery (DAVID)^33,34^. This analysis revealed enrichment for several well characterized processes (Table 2), with the most significant cluster of genes highlighting the role of the mitochondria (Table S4).

**Table 2:** Functional annotation clustering of genes differentially expressed in IPF-F as they adapt to the *in vitro* environment.

Annotation cluster	Gene Ontology (GO) Term Summary	Enrichment Score	Gene Count	P Value
1	Mitochondrion	4.91	161	1.40E-08
2	Proteolysis	4.66	137	1.70E-03
3	ER-Golgi Transport	4.62	31	1.54E-05
4	ER	4.15	131	1.69E-02
5	Membrane-enclosed lumen	3.94	233	7.09E-06
6	tRNA processing	3.41	38	1.60E-04

Functional annotation analysis reveals significant mitochondria involvement in IPF-F adaptation to the *in vitro* environment. Summary of the most significant functional categories altered in the IPF-F as they transition into the *in vitro* environment (Enrichment score greater than 3.0). Fold change and annotations for the highest clusters are found in Tables S4 and S5.

### Comparison of differential expression profiles from IPF lung tissue with the fibroblast specific IPF list identifies cell migration associated signal peptides that are altered by the *in vitro* environment

**Figure 3:**
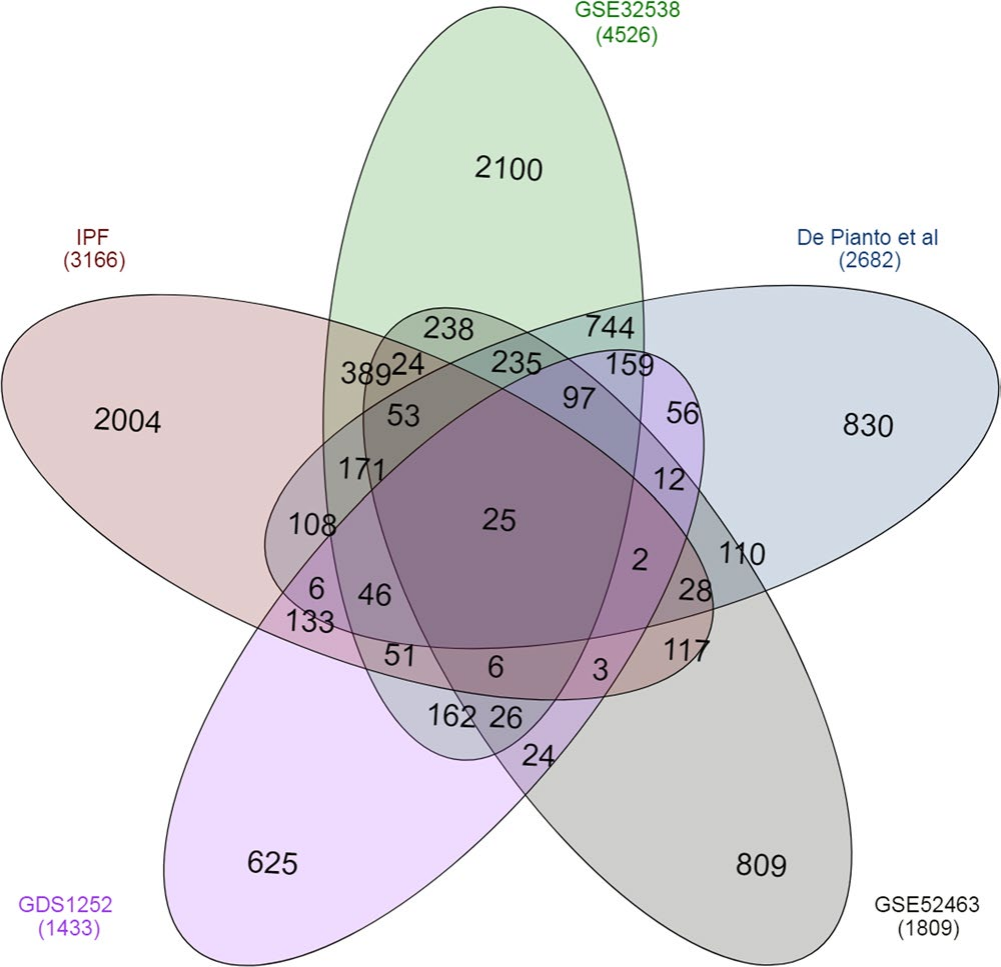
Summary of total number of unigene IDs that are differentially expressed in 4 selected gene lists and the IPF List. The number in the overlaps between each circle correspond to the number of unigene IDs that are found in each list. The IPF list likely represents fibroblast specific genes and any overlap between other lists highlight the fibroblast signature identified in each of the other published lists.

Four publicly available data sets^29–32^ derived from comparisons of global gene expression between IPF and normal whole lung tissue isolations were compared with our IPF-F specific list. For clarity, we will refer to the referenced data sets derived from the literature as the public data and we will refer to our fibroblast specific list as the IPF list. The public data were not reanalyzed, rather these selected lists were compared as originally reported. This comparison resulted in 25 genes that overlap across all five of the reported lists (Fig. 3, Table S6). Functional annotation analysis clustering resulted in two major clusters; a xenobiotic response and signaling peptides. The xenobiotic response is primarily due to a single unigene ID corresponding to nine splice variants of the same cytosolic glucuronosyltransferase and thus is not reported here. The second cluster resulted in the identification of several secreted peptides (Table S6).

**Figure 4:**
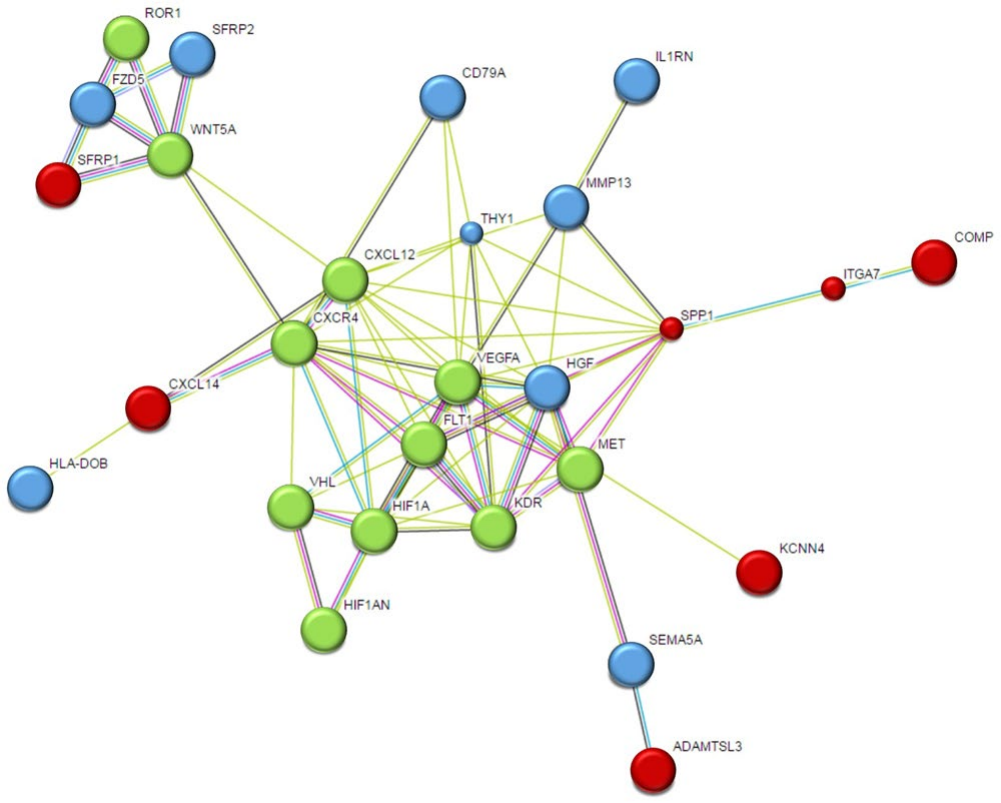
STRING diagram representing protein interaction pathway derived from 25 genes identified in all selected lists and 34 genes found in the IPF list and at least 3 of the 4 published lists. The average local clustering coefficient as reported by STRING is 0.697. Proteins in red are from the all list encompassing 25 genes. Proteins in blue are found in the additional 34 gene list. Proteins in green are STRING predicted functional partners with STRING scores greater than 0.995. This network, among other biological functions, is significantly enriched for proteins involved in cell migration and regulation of developmental pathways.

To capture biologically relevant data, we decided to include genes which were found in the IPF list and which overlapped with at least 3 of the 4 public lists. This expanded analysis resulted in identification of 34 additional genes bringing the total relevant gene list to 59 genes (Table S6). STRING biological pathway analysis of this 59-gene set (Fig. 4, Table S7) highlighted significant pathways corresponding to cell migration and the regulation of developmental processes. In concert with previous findings, some of these genes and their protein partners have been explored as possible biomarkers for IPF (MMP13^35^, CXCL14^36^, VEGFA^37^). A result of particular interest is the CXCR4/CXCL12/CXCR14 axis as our string analysis indicates a possible role of these genes as part of a linker axis for the WNT pathway, VEGF angiogenetic pathway and the HIF1 regulated oxidative stress response. To demonstrate the value of refining the whole lung data sets with our derived IPF list, we performed overlap analysis solely with the data from the public lists. This analysis resulted in 704 genes found in at least 3 of the 4 public data sets (Table S8). STRING pathway analysis and enrichment was carried out as before by removing non-connected nodes resulting in a network of 518 genes with enrichment in biological pathways corresponding to cell adhesion and organ/tissue development (Figure S2, Table S9). Comparison between the two derived STRING pathways demonstrates the significant refinement provided by the inclusion of the IPF list as the average node degree increases from 3.47 to 4.69 and the average local clustering coefficient rises from a loosely connected 0.337 to a highly connected 0.697.

### *In-vitro* gene expression adaptation can be reversed through *in-vitro* challenge by hydrogen peroxide

**Figure 5:**
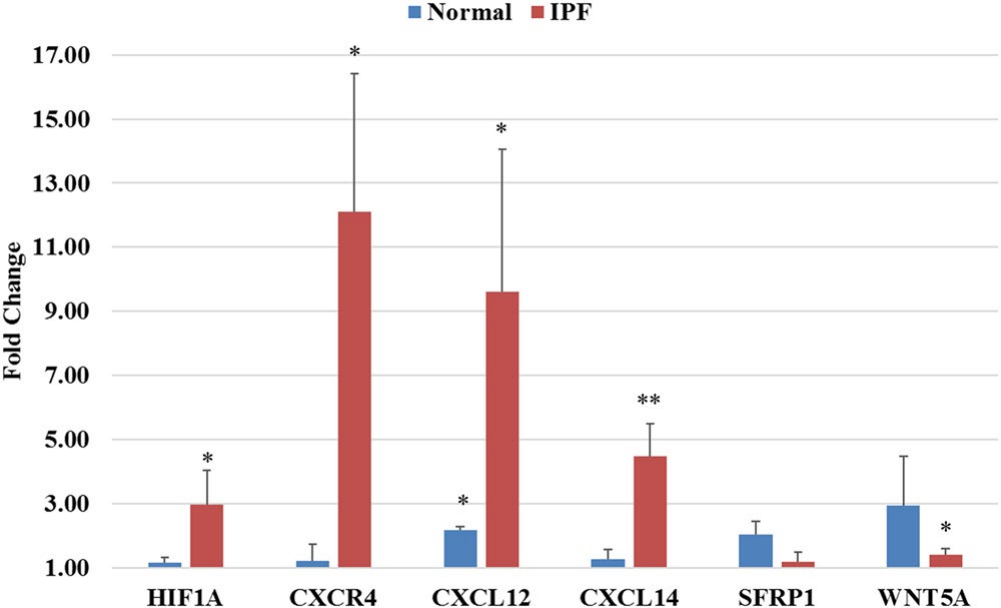
Differential gene expression of select genes after 24-hour 250 µM hydrogen peroxide challenge. Significant changes in gene expression are denoted with *(p < 0.05) or **(p < 0.005). Normal fibroblasts demonstrate minimal response in selected gene panel with significant increase only in CXCL12 gene expression. IPF-F respond with significant increases in all but one gene (SFRP1). Fold changes, delta CTs, and p-values are reported in Table S10 as well as graphical plots of the delta CTs for each gene.

To assess the role of environment, namely the loss of IPF environment, on the *in vitro* behavior of these fibroblasts we challenged IPF fibroblasts with low doses of hydrogen peroxide (250 µM) for 24 hours to simulate the oxidative stress present in the IPF lung. We then examined the expression of six genes with close connections to the CXCR4/CXCL12/CXCL14 axis found in the string pathway (Fig. 5). To measure global cellular response to this oxidative stress challenge we measured expression of HIF1A as it is an oxidative stress inducible transcription factor and present in the string pathway analysis. Additionally, we selected SFRP1 and WNT5A to confirm the predicted relationship between the CXCR4/CXCL12/CXCL14 axis and the WNT pathway. We observed a strong response to this oxidative stress in the IPF fibroblasts (n = 4) with significant increase in expression of the HIF1A gene and an additional 4 genes of the total genes assayed. The lack of significant change in SFRP1 indicates that our oxidative stress model perhaps incompletely simulates the IPF disease environment, however WNT5a significantly increases suggesting that this model accurately reflects the hypothesized interaction in our string pathway. Additionally, the normal fibroblasts (n = 3) show minimal response with only a significant change in expression of CXCL12 (FC 2.18, p = 0.004) indicating a possible increased sensitivity to oxidative stress in IPF-F perhaps through a long-term adaptive mechanism.

### Localization of CXCL14/CXCL12 and CXCR4 within histological samples of IPF lung tissue

**Figure 6:**
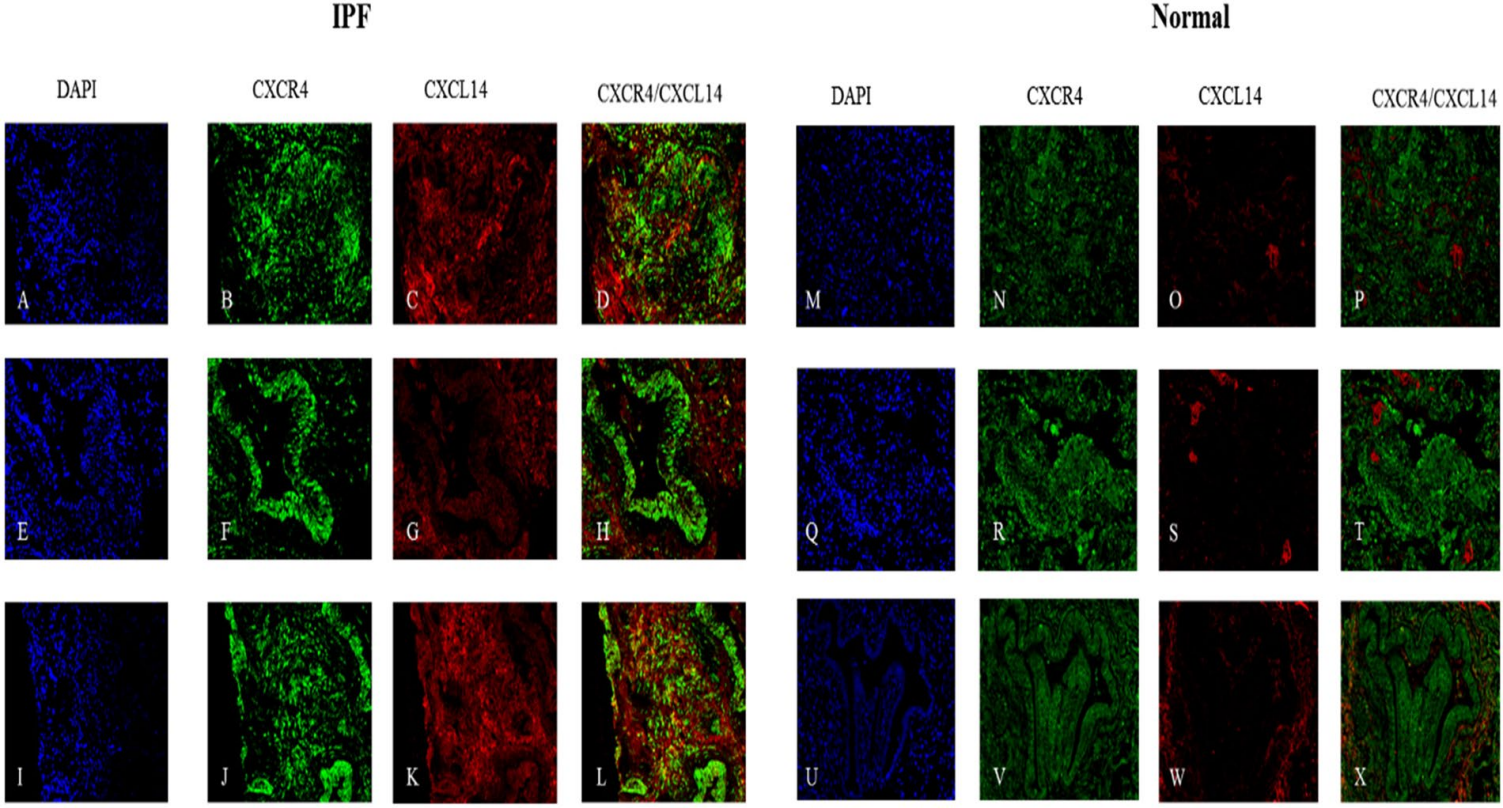
Histological confirmation of the increased expression of CXCL14 in IPF. Representative images of three IPF lung tissue sections (**A–L**) and three normal tissue sections **(M–X**) are stained for the presence of CXCR4 and CXCL14 with dapi counter stain. Increased expression of CXCL14 is observed in IPF only (**C,G,K**). The overlay denoted in yellow identifies regions of co-localization between CXCL14 and CXCR4 in regions of fibrosis (**D,L**). Wide spread CXCR4 is seen in all tissue (**B,F,J,N,R,V**) and specifically in the airways of both IPF (**F**) and normal tissue (**V**). CXCL14 is not seen in the airways of selected tissue (**G,W**).

To determine the localization of CXCL14/CXCL12 and CXCR4 we carried out histological staining of normal and IPF tissue sections from the lower peripheral lobe. Increased expression of CXCL14 was confirmed as seen in all gene lists within the IPF lung (Fig. 6C,G,K). Expression of CXCL14 was not observed in normal lung (Fig. 6O,S,W). Significant expression of CXCR4 was also observed in both the IPF and normal lung sections (Fig. 6B,F,J,N,R,V). Co-localization of CXCR4 with CXCL14 was also evident in regions of dense fibrosis (Fig. 6D,L) in IPF lung tissue. In addition we report pockets of CXCR4 expression that lack co-localization with CXCL14 in IPF lungs (Fig. 6H) which confirms a widely observed trait in normal tissue sections (Fig. 6P,L,N), specfically this phenomon is most distinct in the airways of both normal (Fig. 6N) and IPF (Fig. 6H) sections.

**Figure 7:**
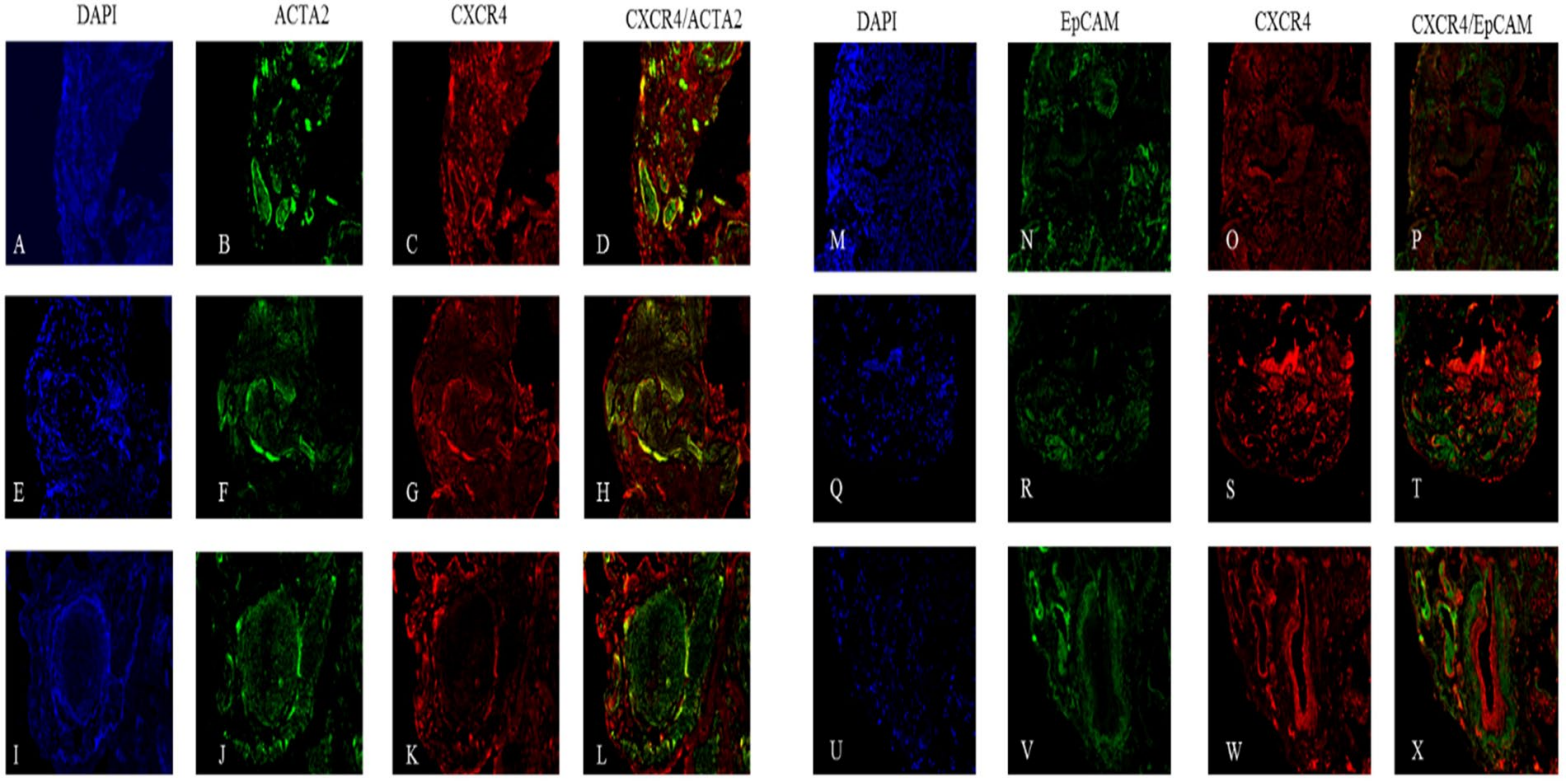
Histological localization of CXCR4 to ACTA2^+^ cells and EpCAM^+^ cells in IPF. Representative images of three IPF tissue sections are stained for the presence of CXCR4 (**C,G,K,O,S,W**). Fibrotic foci stain positive for ACTA2 throughout but only show co-localization (denoted in yellow) with CXCR4 near the peripheral edges (**L**). Areas of dense fibrosis stain show co-localization of CXCR4 and ACTA2 (**D,H**) and the minimal presence of non-CXCR4 co-localized EpCAM (**N,V,R**).

In areas of ACTA2 positive fibrosis in IPF lungs (Fig. 7B,F,J) we observed no expression of CXCR4 within fibroblastic foci (Fig. 7K), however expression of CXCR4 was evident at the periphery of the foci (Fig. 7G) and in areas with either pockets of fibrosis (Fig. 7C) or in less dense wide spread fibrosis (Fig. 7G). Additionally, we identified fibrotic regions where epithelial cells were present (Fig. 7N,R,V). These regions stained positive for CXCR4 expression (Fig. 7O,S,W) however, there was no significant observable co-localization of CXCR4 and the epithelial marker EpCAM (Fig. 7P,T,X).

**Figure 8:**
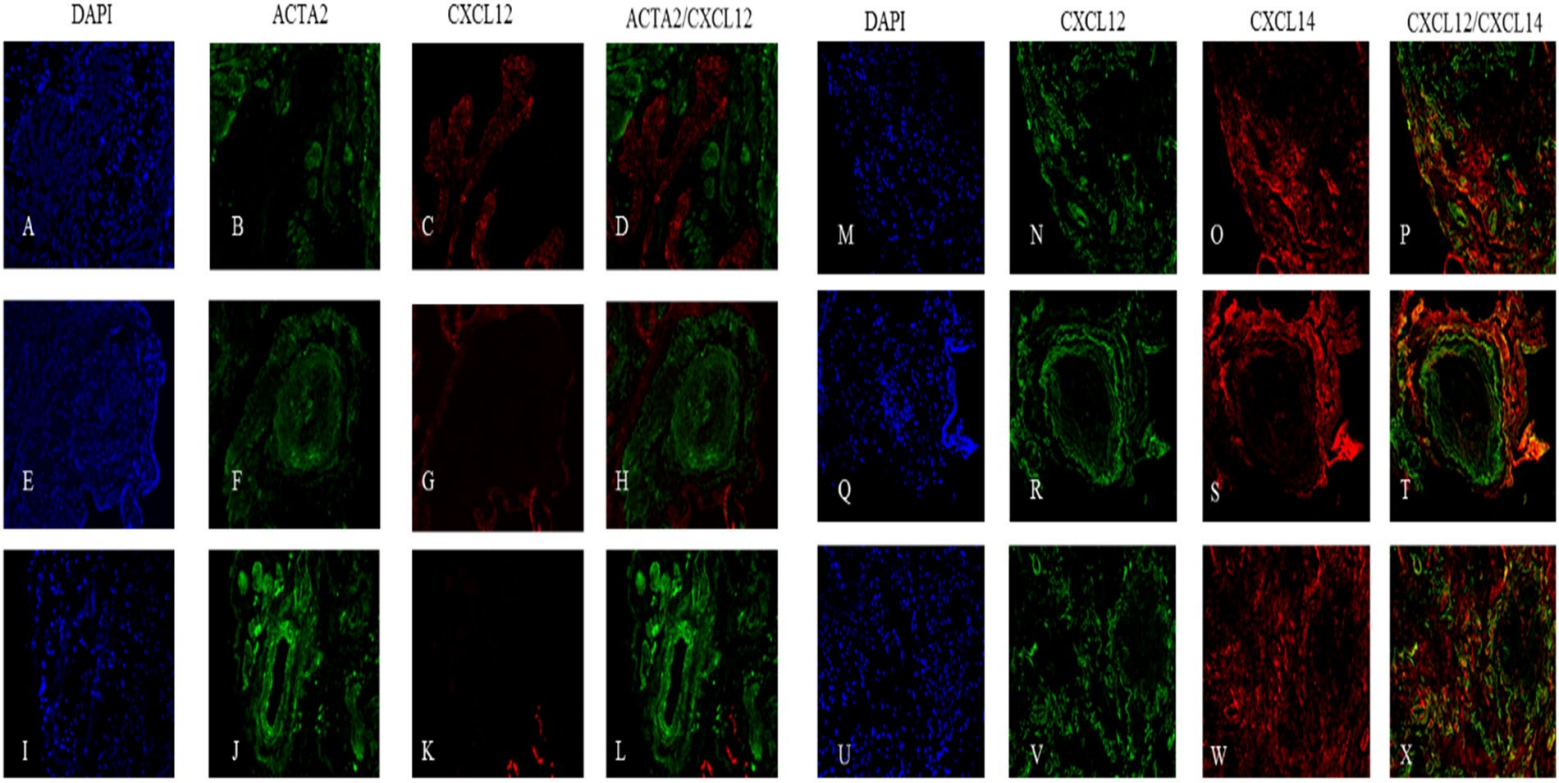
Histological localization of CXCL12 and CXCL14 in IPF. Representative images of three IPF tissue sections are stained for the presence of CXCL12 (**C,G,K,N,R,V**), ACTA2 (**B,F,J**), and CXCL14 (**O,S,W**). CXCL12 is absent in foci (**H,T**) or areas of significant fibrosis (**L**), sparingly distributed in lower fibrosis regions (**S**), and highly present in airways (**D**). No co-localization of ACTA2 and CXCL12 is observed (**D,H,L**), with minimal co-localization of CXCL12 and CXCL14 seen in diffuse fibrosis (**P,T,N**).

We also report no observable co-localization between ACTA2 and CXCL12 in selected IPF tissue sections (Fig. 8D,H,L), rather we see predominant localization of CXCL12 within IPF airway epithelium concurrent with previous reports^8^ (Fig. 8C,D). Finally, we report minimal co-localization of CXCL12 and CXCL14 on the periphery of fibrotic foci (Fig. 8T) and in areas of diffuse fibrosis (Fig. 8P and X). A summary of all findings is listed below (Table 3).

**Table 3:** Summary of Histological Findings.

Tissue Characterization	CXCR4	CXCL14	CXCL12	ACTA2	EpCAM
IPF-Foci	−	−	−	++	−
IPF – Regions of Fibrosis	++	++	+	++	+
IPF – Airways	++	−	++	−	not reported
Normal Tissue	++	−	not reported	not reported	not reported
Normal –Airways	++	−	not reported	not reported	not reported

Summary of the presence or absence of the protein target within the tissue sections reported in Figs 6–8. Moderate to high presence of the protein is denoted with ++, low levels are denoted by + , and the absence of the protein is marked by −. Any proteins not specifically reported in the results are designated “not reported”.

## Discussion

Analysis of whole lung tissue in IPF has resulted in several important findings identifying many of the key underlying processes in IPF; however, identifying disease specific processes that are primarily related to and/or regulated by the fibroblast is difficult in the whole tissue background. Our hypothesis is predicated on the idea that some fibroblast genomic adaptation to the disease environment is lost through the process of isolation and tissue culture. To address this issue, we documented the differential expression profile of IPF and normal fibroblasts from isolation and during culture. This unique approach demonstrated that there are a number of cellular pathways that are altered by the process of *in-vitro* culture in the IPF-F. The findings, summarized in Fig. 1, indicate that there are many genes in the fibroblast transcriptome that are altered by the tissue culture environment (*in vitro*) regardless of the *in-vivo* origin, IPF or normal. This analysis also highlights the many genes whose transcriptional control is directly influenced by the IPF environment and are separate from the intrinsic propensity of the fibroblast itself.

To demonstrate the utility of this approach we first compared four broad IPF whole lung tissue profiles to find overlapping processes within these lists. As expected, there is substantial concordance within these publically available data lists resulting in more than 700 genes overlapping (Table S8). Refinement of this list to interconnected proteins results in a network of 518 genes with a loose clustering coefficient of 0.337. This list was significantly enriched for biological pathways that control cell adhesion and organ/tissue development (SF2, Table S9) which are of interest in IPF disease biology, however with such a broad loosely connected network, identification of a small number of highly significant genes is difficult without further refinement.

By comparing our fibroblast specific list to the same four broad IPF whole lung tissue profiles, we identified a narrow subset of 59 IPF specific genes that we believe are indicative of the environmental impact on the fibroblast phenotype in disease. Many of these genes correspond to signaling peptides indicative of a dynamic crosstalk between the IPF environment and the IPF fibroblast. Using a smaller focused set of genes, we generated a string pathway which resulted in significant enrichment for biological pathways that control cell migration and developmental processes (Fig. 4, Table S6). As compared to the 518-gene network, our refined list resulted in a clustering coefficient of 0.697 indicating the interdependence and significance of each node within the network.

Our data is in line with previous reports of several IPF pathogenic pathways, namely the WNT pathway^38,39^ (e.g. FZD5, SFRP1, SFRP2) and migratory associated processes^40–42^ (e.g Thy1, MMP13, ADAMTSL3) that respond to the IPF environment. Through our STRING analysis (Fig. 4) we observed the central role that the CXCR4/CXCL12/CXCL14 axis plays in the connection of these pathways. Of particular note is the observation that these three genes are all downregulated through the *in-vivo* to *in-vitro* transition of the IPF fibroblast (Table S2). We hypothesize that expression of these genes and this axis is environment dependent and that the change in environment brought on by the *in vitro* transition resulted in their reduced expression. In addition, we theorize that this transition may be reversible, for example by placing these IPF fibroblasts in an *in vitro* environment with the simulated stress of an IPF lung.

To investigate this hypothesis, we induced oxidative stress by exposure to a 24-hour sub lethal dose of hydrogen peroxide. This resulted in the predicted increase in gene expression of the entire CXCR4/CXCL12/CXCL14 axis, a response which was exclusive to the IPF fibroblasts and not observed in normal fibroblasts, indicative of an increased sensitivity of the IPF fibroblast to oxidative stress. These observations warrant future and further exploration as they may be important factors in the progression of IPF. Our next interest was the localization of this cytokine axis within the IPF lung.

Given the data discussed thus far we predicted that the IPF lung would be enriched for CXCR4^+^ fibroblasts and that CXCL12 and CXCL14 would co-localize with these same fibroblasts. Histological examination of the IPF lung did indeed demonstrate wide spread CXCR4 receptor expression and that many ACTA2^+^ cells co-expressed CXCR4; however surprisingly, expression was not exclusive to ACTA2^+^ regions. Of particular interest was the observation that there was little to no CXCR4 within the fibrotic foci, while at the leading edges and in areas of less dense fibrosis CXCR4 is almost uniformly expressed. These data indicate that CXCR4 may be important in initially aiding migration and recruitment of fibroblasts or proliferation but may no longer be required once they have settled into the signature foci.

The most intriguing data arises from the CXCL12/14 localization. Recent studies debate the agonistic/antagonistic relationship between these two ligands (CXCL12/14) and the receptor CXCR4^43,44^. We observed minimal co-localization of these two ligands in areas of less dense fibrosis. In spite of the widespread observed presence of CXCR4 and CXCL12 (Figs 6 and 8) in these lungs we observed that that the CXCL12 ligand tends to localize in the airway epithelium, as previously reported by Andersson-Sjoland *et al*., while also reporting no co-localization of CXCL12 and ACTA2. However, we did observe CXCR4 and CXCL14 co-localization within regions of ACTA2 expression (Figs 6 and 7) outside of the fibrotic foci. The nature of these foci is a central question in our understanding of the disease process. Progression of the disease is marked by an increase in the number and size of these fibroblast aggregates which appear to be sites of active fibrosis^45^. It has been recently demonstrated that ACTA2 expression is variable within the lung and that there is an inverse relationship between relative ACTA2 expression and the senescent phenotype^46^. Furthermore, *Shafer et al*. confirms that the senescent fibroblast is a driver of ECM secretion and increasingly found within the foci throughout disease progression. Given that the currently understood role of CXC chemokines in IPF is as a mechanism of recruitment and vascular remodeling^47^, and our findings that the primary receptor for CXCL14 is outside of the fibrotic foci, these data suggest that CXCL14 may serve as a signal for the recruitment and activation of CXCR4^+^/ACTA2^+^ cells.

The role of CXCL14 in fibroblast biology is poorly understood. However CXCL14 has been identified in cancer associated stromal fibroblasts as a promoter of migration and ERK-dependent proliferation^48,49^. Prostate tumors develop rapidly and display increased angiogenesis in the presence of CXCL14 overexpressing cancer associated fibroblasts^48,50^. We speculate that in the IPF lung the CXCL14/CXCR4 interaction may be a driving force for the recruitment or migration of fibroblasts to the sites of fibrosis and that once the foci form, there is decreased need for this axis and therefore a decrease in expression. This hypothesis is further supported by recent studies into both CXCL12 and CXCL14. Like CXCL12, CXCL14 is over expressed in whole lung tissue of IPF patients^19,29,30,51^ and also found at higher levels in the serum of IPF patients^36,52^ strengthening its potential role as a recruitment mediator. *In-vivo* studies targeting CXCL12 in lung fibrosis models have overall been inconclusive, however they have demonstrated some success in the slowing of disease progression^53,54^. We theorize that inclusion of CXCL14 may reveal a complementary role for this ligand in the axis that when addressed may effectively target this pathway. Further studies into the significance of the co-localization of CXCL14/CXCR4/ACTA2 within the IPF lung needs to be performed, however, we speculate that it may be possible to slow or inhibit fibroblast migration and activation by pharmacological intervention directed at the CXCR4/CXCL14 complex.

## Conclusions

IPF is a progressive disease that presents in acute, progressive, and stable variations based on many yet unknown environmental and genetic factors^55^. There is general agreement that therapeutic intervention in fibrotic disease is complicated by the heterogeneity of both the IPF lung and the disease as a whole. Our study highlights an added layer of complexity which further can cloud or even misdirect our understanding of fibroblast biology. Our unique approach highlights the transcriptomic changes observed through the first weeks of tissue culture that has enabled us to identify a subset of genes not previously reported as significant in IPF.

A potential limitation to this study may emanate from what we have termed as the initial fibroblast population isolated from the IPF lung. Criticism of this technique, which is widely employed in other fields of study, may call into question the purity of the population at initial isolation. We addressed this concern in three ways: first, we used previously published data sets and string pathway analysis to refine the large number of genes in the IPF list. Second, we confirmed expression in an *in-vitro* fibroblast culture model both before and after challenge. Finally, we identified co-expression of our most interesting gene, CXCL14, with ACTA2 *in vivo* in areas of fibrosis. Identification of CXCL14 as a gene of interest in IPF has been recently been reported^17,56^, however, to our knowledge, this is the first time that CXCL14/CXCR4/ACTA2 has been demonstrated together in the IPF lung and attributed to disease. This observation has not been previously described, which we hypothesize is due to the environmental limitation inherent to *in-vitro* tissue culture. We therefore suggest that our data set and model system can be applied to other IPF associated pathways to help identify potential therapeutic targets.

In conclusion, we have taken a wide range of novel and published data and focused it through a model system that reduces the confounding effects of the traditional *in-vitro* tissue culture model. In doing so we identify a potential role for CXCL14 in IPF within the CXCL12/CXCL14/CXCR4 axis that is in accordance with our data and four published data sets. As a soluble ligand that is upregulated in IPF, CXCL14 serves as a candidate for further study that may also lead to a therapeutic intervention. Finally, we propose that further application of this data set may result in the identification of novel processes of interest in the IPF lung.

## Materials and Methods

### Donor Consent and Internal Review Board Approval

IPF lung tissue was obtained through Inova Fairfax Hospital (VA). All normal control lungs were obtained through the Washington Regional Transplant Community (WRTC). Appropriate written informed consent was obtained for each patient and donor by Inova Fairfax hospital and the WRTC. This study was approved by the Inova Fairfax Hospital Internal Review Board (IRB #06.083) and the George Mason University Human Subject Review Board (Exemption #5022). All experiments were performed in accordance with relevant guidelines and regulations. Additionally, no organs/tissues were procured from prisoners.

### Specimen procurement/dissection and cell culture

The primary fibroblasts used in this study were isolated from human lungs procured in the operating room within minutes of explantation. The lungs were oriented from apex to base and all samples used in this study were taken from the peripheral lower lobe of the lung. Fibroblasts were isolated from the lung tissue of 8 patients with advanced IPF (IPF-F) and 4 normal (Normal-F) controls as previously described^17,57^. Briefly, samples were dissected into 1–2 mm^2^ pieces and subjected to enzymatic digestion in 0.4% collagenase P (Roche, Indianapolis, IN) complete media (Dulbecco Minimal Essential Media (DMEM) containing 10% fetal bovine serum (FBS), penicillin (100 I.U/ml), streptomycin (100 MCH/ml), amphotericin B (0.25 M.C.G./ml P/S/A) and 0.1% DNase1, at 37 °C and 5% CO_2_ for 2 hours. The resulting material was passed through sterile cell filters (40, 100 µ nylon mesh) to remove undigested tissue and remaining cells were pelleted by centrifugation at 1000 g for 5 min. The pelleted cells were then suspended in complete media and seeded onto tissue culture treated plastic at 37 °C and 5% CO_2_ for 45 minutes. The attached fibroblast population was vigorously washed with PBS to remove any unattached cells. P0 cell populations were extracted for RNA immediately. P3 populations were continued in culture and maintained in complete media at 37 °C and 5% CO_2._ All demographic information for the patients is listed below (Table 4).

**Table 4:** Lung Donor Demographics.

Demographic	IPF (n = 8)	Normal (n = 4)
Age (Years)	64.2(±4.5)	50.8(±9.5)
Gender	Male: 7	Male: 2
	Female: 1	Female: 2
Smoking History	Prior: 6	Prior: 0
	None: 2	None: 4
Race	Caucasian: 8	Caucasian: 4

### Microarray Analysis

RNA was extracted from IPF-F and Normal-F fibroblasts at P0 and P3 using a Qiagen RNeasy kit (Qiagen, Valencia, CA) and DNase treated using DNase free (Ambion, Austin, TX). The quality and quantity of the RNA was determined using RNA 6000 nanochips and an Agilent Bioanalyzer. For analysis, 1 µg of RNA was amplified and amino-allylated using the MessageAmp II aRNA kit (Ambion). All microarrays were carried out by the Duke Institute for Genome Sciences and Policy, Microarray Facility using HO36K human chip representing 33,791 transcripts from the Ensembl Human Build (BI-35C) including 22,169 unique genes (Operon Human Oligo set V4). Using two color amino-allylated amplified RNAs: cy5 corresponding to patient and normal samples, and cy3 for Stratagene human reference RNA. Microarray results were confirmed by QPCR analysis using a random selection of 11 genes (Supplemental Fig. 1).

The resulting genepixs files were analyzed using the TM4 software suite (ExpressConverter V1.0, MIDAS, MEV) and normalized by locally weighted linear regression (lowess)^58^ Normalized, filtered (percent filter cut-off 60%) data was analyzed using statistical analysis of microarray (SAM)^34^. Any resulting transcript without a corresponding Unigene ID was not considered in this analysis. Additionally, some unigene IDs did not have a corresponding ID in the Database for Annotation, Visualization, and Integrated Discovery as noted in the discrepancy between Unigene and David IDs below. Table 5 describes the number of genes identified for each comparison as well as the filters (Delta) and false discovery rates (FDR) utilized to identify those gene alterations.

**Table 5:** Differential Expression Gene Count Across All Subpopulations Analyzed.

Comparison	Unigene IDs Identified	Number of David IDs	Delta	FDR
IPF P0 - P3	3323	2889	2.49	0
Normal P0 - P3	1287	1118	1.85	0
IPF P0 - Normal P0	1398	1184	2.26	0
IPF P3 - Normal P3	0	0	0	0

### Bioinformatics

Ontological analysis and classification of the genes identified in each analysis was carried out using the Database for Annotation, Visualization, and Integrated Discovery (DAVID) as previously described^33,34^. Functional categories were clustered using the Functional Annotation Clustering tool, and clusters with a score of greater than 3.0 were selected for discussion. Protein-protein interactions were obtained using STRING (Search Tool for Retrieval of Interacting Genes/Proteins) database. Interactions were parsed in three steps. Initial input of 59 proteins corresponding to differentially expressed genes were entered into STRING. All genes with predicted associations were curated manually and any closely linked non-associated proteins were also selected. The second step was simply the redefined string network that only included the manually curated proteins. Finally, predicted functional partner network linker nodes (string score > 0.995) were added to complete the network.

### Hydrogen Peroxide Challenge

Fibroblast populations isolated as described above were seeded in 6-well falcon tissue culture plates for 24-hours in complete media. After this attachment cells were placed in serum free DMEM for 24-hours and then challenged with serum free DMEM containing 250 µM hydrogen peroxide. Cells were then harvested for RNA analysis as described above. Assays were carried out on cells in passages 4–7. Two of the four IPF cell lines selected for this assay were derived from the pool of cells isolated for the microarray analysis and another two cell lines were derived from IPF donors not included in the microarray data set. All non-disease cell lines challenged by hydrogen peroxide were derived from normal donors in the microarray dataset.

### Quantitative Real Time PCR (QPCR) Analysis

All QPCR was carried out using cDNA generated from 1 µg of total RNA using iScript cDNA synthesis kit (Bio-Rad, Hercules, CA) and Quantifast SYBR Green PCR Kit (Qiagen). QPCR was carried out in triplicate and normalized to 18 S expression levels using the delta delta CT method^59^ (Primer sequences and efficiency Table S12). Primers were tested for single target specificity prior to use in experiments through melting curve analysis. Primer efficiency was generated through generation of standard curve from Universal Human Reference RNA (Agilent). QPCR of standard curves for each primer were also carried out in triplicate. Statistical analysis was performed using Microsoft Excel one-way paired student T-tests. Expression fold differences with p values less than 0.05 were considered significant.

### Immunocytochemistry

Formalin fixed paraffin-embedded IPF and normal tissue sections (3–5 µm) were deparaffinized by serial washings in xylene, 100, and 90% ethanol prior to rehydration in nuclease free water. Antigen retrieval was performed by boiling in sodium citrate buffer (pH 6). Slides were blocked for 1 hour in 1% BSA prior to overnight incubation with primary antibodies: anti-actin smooth muscle (Thermo #PA5–16697), EpCAM (Cell signaling #14452), CXCR4 (Santa Cruz #sc-53534), CXCL12 (Santa Cruz#P-159X), CXCL14 (Thermo# PA5-528820). Primary antibody incubation was followed by 1 hour secondary incubation in Alexa Fluro 594 or 488 conjugate (Cell signaling #8890 and #8889). Counter staining with DAPI was performed prior to imaging using Life Technologies EVOS FL. All antibodies were tested for cross reactivity prior to use and secondary antibodies were added in the absence of the primary antibody to confirm lack of non-specific binding. No cross reactivity was identified and minimal background florescence was detected at very high light levels which were not used during imaging. All images were taken from three different IPF and three different normal lung sections derived from the lower peripheral lobes. Select images are representative of whole section observations.

### Availability of data and materials

The microarray data sets used in this analysis are available in the Gene Expression Omnibus repository, https://www.ncbi.nlm.nih.gov/geo/query/acc.cgi?acc=GSE87175.

## Electronic supplementary material

Supplmentary Information
